# Design and development of a disease-agnostic remote patient monitoring typology and associated tools

**DOI:** 10.1371/journal.pdig.0001595

**Published:** 2026-07-31

**Authors:** Ibukun-Oluwa Omolade Abejirinde, Vanessa Kishimoto, Janette Brual, Kaylen J. Pfisterer, Nishath Uddin, Taylor Pratt, Cathleen Fleury, Azza Osman, Onil Bhattacharyya

**Affiliations:** 1 Institute for Better Health, Trillium Health Partners, Mississauga, Ontario, Canada; 2 Institute for Health System Solutions and Virtual Care, Women’s College Hospital, Toronto, Ontario, Canada; 3 Social and Behavioural Health Sciences, Dalla Lana School of Public Health, University of Toronto, Ontario, Canada; 4 Centre for Digital Therapeutics, University Health Network, Toronto, Ontario, Canada; 5 Systems Design Engineering, University of Waterloo, Waterloo, Ontario, Canada; 6 Department of Family and Community Medicine, University of Toronto, Toronto, Ontario, Canada; Maastricht University Cardiovascular Research Institute Maastricht: Universiteit Maastricht Cardiovascular Research Institute Maastricht, NETHERLANDS, KINGDOM OF THE

## Abstract

Remote patient monitoring (RPM) expanded dramatically during the COVID-19 pandemic and continues to be implemented. However, no standardized method for classifying them exists which has implications for comparative evaluations. Our goal was to design and develop a disease-agnostic RPM typology tool as a first step towards a standardized approach for understanding and implementing RPM programs for different clinical use-cases. Guided by the Knowledge-To-Action framework, we conducted a rapid review of RPM programs from Canada, the United States, Europe, the United Kingdom, and Australia that were used to manage diabetes, chronic obstructive pulmonary disease, congestive heart failure, hypertension, and COVID-19. We identified 87 articles to define common characteristics of real-world RPM interventions to enable comparison across different programs through pattern recognition, information mapping, and sensemaking. We extracted data with a macros-enabled Excel template. Design sessions with key stakeholders (researchers, clinical advisors, provincial RPM managers, and patient partners) provided iterative feedback on the typology and we defined a glossary of characteristics. The 12 most reported characteristics of RPM programs (including size, resources, monitoring team, data flow, alert protocol, workflow, and equity considerations) were clustered into four domains. Integration and equity domains were recognised as ideal aspirations of all RPM programs. Technology and touch domains were considered to exist on a spectrum from low-to-high- neither inherently superior to the other. 16 distinct RPM typologies were expressed through a 4x4 matrix (i.e., high or low on each of the four domains). Using this tool can inform insights on program maturity, implementation, and continued investments in RPM. We anticipate this typology will help new initiatives have greater potential to be robustly evaluated and sustained to scale. Future directions include further validity testing and exploring feasibility to systematically categorise real-world RPM programs with this tool.

## Author summary

Remote patient monitoring programs allow care teams to manage patients from a distance. But understanding how their features and outcomes compare to each other is a challenge because there many different types of such programs. We designed a tool based on 87 research articles and feedback from different people to define 12 core elements of remote monitoring programs divided into 4 categories. Our tool can be used to map different programs, track their implementation features as they evolve, and compare programs like-for-like. This allows us to learn from what works best even when the programs are designed to support different illnesses. Our goal is for this tool to help decision makers better evaluate these programs so they can be more smartly designed to support patients most effectively. It can also help researchers and funding agencies that design or evaluate remote monitoring programs in determining areas that may require additional investments.

## Introduction

### Background

With an increasing burden of multimorbidity and higher demands for care under constrained health system resources, remote patient monitoring (RPM) has garnered interest in its potential to enhance healthcare access and coordination of care facilitated at a distance [1,2]. They are valued for enabling monitoring of a patient’s clinical conditions outside of in-person or acute care settings, facilitating timely detection and responsive clinical action at the deterioration of illness [3]. Health system shocks in the wake of the COVID-19 pandemic resulted in growing demands on a reduced workforce, and patients with complex needs required more hands-on support and wraparound care [4]. Preference has also increased for people to age at home or in their communities [5–9]. Yet if not properly managed, there is a risk that frequent hospitalizations and emergency department (ED) visits will continue to be the norm [10–12]. Digital health solutions may be well positioned to support management of chronic conditions with improved clinical outcomes [13,14]. However, not everyone who desires to be monitored remotely has access to these technologies and attention must be paid to bridge the digital divide and inequitable access to care [15–17]. For example, data shows that 41% of 12,052 surveyed Canadians (20% of whom are 65 + years) have an unmet demand for RPM programs and services to manage chronic conditions [18].

Traditionally, RPM has been designed and implemented with a condition-centric approach (i.e., to support specific clinical needs such as surgical transitions, diabetes, heart failure) separately, rather than as a model of care that centers the patient throughout their care needs. As such, limited attention has been given to variations in technological dependence, clinical workflows, patient populations, and resources - making it difficult to (i) understand which programs are effective and why, (ii) assess their stage of technological implementation or maturity, and (iii) identify key features that characterise good RPM solutions. A realist review that aimed to identify the factors that may influence effectiveness of RPM interventions presented six theories that can inform strategies to ensure the right kind of program model for different targeted populations [19]. These include targeting population focus, responsiveness of escalation parameters, enhancing self-management, and strengthening coordinated care. Evidence from systematic reviews show widely varying outcomes from RPM programs which has been explained as being due to variations in patient selection, clinical models, implementation strategies, and features of the technology [20,21]. When compared to usual care, a recently published pooled analysis of 58 chronic disease RPM programs showed reduced mortality and improved physiologic measures, but higher risk of hospitalization [22]. However, the study did not establish if these were avoidable hospitalizations, since RPM is intended to result in just-in-time interventions that prevent mortality. Furthermore, although digital health innovations are touted as being able to equitably improve access to care, the COVID-19 pandemic revealed that technological innovations in healthcare may perpetuate or create inequities [23,24]. In particular, communities who are structurally marginalized by age, race, gender, geography, disability, amongst others, are likely to experience an increased digital divide due to intervention-generated inequalities [25]. Similarly, RPM programs can also inherently restrict access due to equity related factors [17,26].

Between 2021 and 2022, the provincial health bodies in Ontario, Canada - the Ontario Ministry of Health (MOH) and Ontario Health (OH), funded the implementation of more than 40 RPM programs across the province with diverse clinical use cases including for COVID-19 management, chronic conditions, alternate levels of care, and surgical transitions. These were implemented by multiple healthcare organizations using different technology vendors. To understand which programs were better positioned to scale and were on track to advance the quintuple aim of healthcare (i.e., cost effectiveness, positive health outcomes, good patient and provider experiences, and equity [27]), the MOH and OH sought to evaluate this heterogenous group of RPM programs. While such an evaluation could guide future planning and investments in digitally enabled healthcare and inform knowledge on the impact of RPM, the heterogeneity of the programs – a situation not unique to the Ontario cohort, posed an initial challenge. To ensure a like-to-like comparison of programs despite varying clinical pathways, implementation timeline, enrolled users, etc. the body of evidence and decision-making on RPM needed a common point of reference. Recognising this challenge and in the absence of an existing solution, a research team at the Centre for Digital Health Evaluation (CDHE), Women’s College Hospital Institute for Health System Solutions and Virtual Care (WIHV) decided to develop a novel typology of RPM programs.

The rationale for developing a typology is that a disease-agnostic categorisation of RPM that goes beyond clinical use cases and spans the full spectrum of care - from self-management to active management - can advance thinking around the design, implementation, and delivery of RPM programs, and address the high variation in effectiveness evaluations. In this paper, we describe the development of such a first-of-its-kind tool which provides a systematic way to assess, compare, and potentially guide the implementation and evaluation of remote care interventions.

## Materials and methods

### Study design

Two rapid reviews of literature on RPM programs supplemented by expert consultation from clinicians, health system implementers, and patient evaluators constituted our research methodology. This approach aligns with the knowledge creation phase of the Knowledge to Action (KTA) Framework [28], which focuses on producing new or synthesizing existing knowledge in a way that is practical, relevant, and accessible for real-world implementation. A similar methodological approach has been used to develop other program typologies; for example, on service models for inpatient mental health [29], allowing users to categorise healthcare programs in a standardized way while capturing variation. The KTA knowledge creation phase comprises three main stages which are mapped below to the steps we followed in developing the RPM typology: knowledge inquiry (Stage 1), knowledge synthesis (Stage 2), and the creation of knowledge tools and products (Stage 3). For distinction between the approach and the outcome, we report stages 1 and 2 under the methods section and stage 3 in the results section of this paper.

### Stage 1. Knowledge inquiry: Rapid review of literature

Driven by the need identified by OH and MOH, the aim of Stage 1 was to establish the knowledge gap and scope of inquiry by reviewing documented RPM programs from the literature. This was preceded by a non-systematic scan of documents on the funded RPM programs in the Ontario context which described to varying levels of detail their programs and features. This activity allowed us to familiarise ourselves with the variety of features that could be presented in real-world programs before systematically reviewing the literature and identifying characteristics relevant for classifying programs. Next, we carried out two rapid literature reviews in 2022 – one focusing on RPM programs for chronic diseases, and the other on COVID-19 (see S1 Table).

#### Chronic disease literature review.

**Fig 1:**
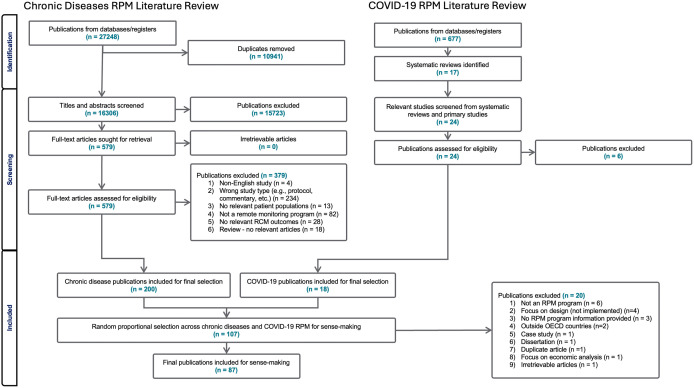
PRISMA diagram for combined chronic disease and COVID-19 literature reviews. A final 87 publications were included for sense-making to define characteristics common across RPM programs, which were subsequently reviewed by an expert panel.

The rapid review on RPM for chronic diseases used search criteria with variations of remote monitoring, and included related and overlapping terms like telemonitoring, telemedicine, mHealth, apps, eHealth, and virtual care. The articles were limited to four chronic conditions, specifically chronic obstructive pulmonary disease (COPD), heart failure (HF), Diabetes (type 1 and 2, gestational diabetes) and hypertension - conditions where RPM has been most widely used. Articles were limited to Canadian and International peer-reviewed articles published between 2017–2022 and in the English language. The search strategy was applied to Embase and Medline. From an initial 27,248 identified articles, 200 were included (Fig 1)

#### COVID-19 literature review.

The search criteria for COVID-19 RPM programs used similar search terms (remote monitoring, telemonitoring, telemedicine, mHealth, apps, eHealth, and virtual care), with a specific focus on its use for people diagnosed with or suspected of COVID-19. Study types included peer-reviewed publications between 2018–2022 and published in the English language. Articles were identified on Google Scholar and PubMed databases. Specific search terms included *“Remote patient monitoring” AND “COVID-19”; “Telemonitoring” AND “COVID-19”;* and *“Telemedicine” AND “Remote” AND “COVID-19.”* From an initial 677 identified articles, 18 articles were included (Fig 1).

#### Final selection of articles across chronic diseases and COVID-19 for sensemaking.

Our intention was to select approximately 110 articles (20 publications per disease category- four chronic conditions and COVID-19, with an additional 10 for multi-disease RPM programs) from the 218 total identified, using non-probability quota sampling, a strategy to ensure diversity and relevance when managing large bodies of evidence [30,31]. Because publications were not evenly distributed across the five disease categories, we defined an initial batch of 107 papers through random proportional selection. Next, we applied one final eligibility criterion, limiting articles to those arising from or pertaining to an Organisation for Economic Co-operation and Development (OECD) member country, as these countries’ health performance indices are commonly used to benchmark global health system policies and reform. Through full-text reading, we further identified and excluded 20 papers that did not meet our inclusion criteria. A total of 87 publications were eventually selected to inform sensemaking and pattern recognition in stage 2 (Fig 1).

### Stage 2. Knowledge synthesis: Information mapping and sensemaking

From the 87 articles identified in the two rapid reviews (stage 1) the research team held meetings to discuss a random selection of 10 articles, going over the program features reported in each paper, specifically those unrelated to the clinical use case of the RPM program. We created a running list of these characteristics through an information mapping exercise, identifying up to 20 information categories by which RPM programs were reported (see Table 1). To extract data from each article into an Excel spreadsheet against these categories, there was a need for the research team to establish two things: (i) the meaning of each category such that it could be easily communicated and uniformly understood, and (ii) the different ways in which information on each category could be reported across RPM programs, such that there was a limited range of options to pick from. For example, to avoid an unmanageable number of choices for the category ‘Program size’ we defined 5 sub-categories- small, medium, large, scaled-up, and unknown, each with its own definition. Establishing these early on ensured coherent interpretation and systematic extraction during the information mapping process. In addition to biweekly meetings between the research team and MOH and OH program teams, we also convened a 6-person expert panel comprising three patient partner evaluators (who have experience with chronic diseases, health service delivery or RPM programs) and three physicians who are experts in virtual care. Collectively these advisors contributed to the sensemaking exercise by providing feedback on definitions, relevance and clarity of each characteristic, and its applicability for classifying diverse RPM programs. The patient partner evaluators were particularly invaluable in ensuring every definition was clear and easy to understand (commonly dubbed ‘lay language’) to someone unfamiliar with the technical details of how RPM programs are designed or function. These series of meetings with the expert panel, resulted in the development of a dictionary or glossary that captured all definitions and sub-options for each RPM category. Somewhat like a “code book”, this was iteratively refined throughout the project as we extracted more data, expanding our RPM vocabulary and encountering new pieces of information from articles. Where information about a specific characteristic was unavailable, we labelled it as ‘unknown’.

**Table 1:** Initial list of information categories on RPM programs and those that were subsequently excluded.

Initial information categories	Was the category excluded from the final list (Yes/No)?
1. Country of implementation	Yes
2. Patient population (i.e., clinical condition being monitored)	Yes
3. Hardware volume (i.e., the number of individual devices a user had to interact with to fully use the RPM system)	Yes
4. User dependency profile (i.e., the extent to which a user requires assistance from a third party for day-to-day use of the program)	Yes
5. Data collected (i.e., the type of vital measures and indicators that were monitored such as temperature, respiration rate, and medication intake)	Yes
6. Monitoring location (i.e., where the monitoring team is located- in the hospital or in the community)	Yes
7. Program size (i.e., the number of people reported to have enrolled in the program)	Yes
8. Size of monitoring team (i.e., number of persons involved in monitoring and or responding to health status signals)	Yes
9. Patient risk profile (see Table 2 for definition)	No
10. Vendor information (see Table 2 for definition)	No
11. Device ownership (see Table 2 for definition)	No
12. Alert protocol (see Table 2 for definition)	No
13. Data entry modality (see Table 2 for definition)	No
14. Frequency of manual data entry (see Table 2 for definition)	No
15. Data access (see Table 2 for definition)	No
16. Availability of monitoring team (see Table 2 for definition)	No
17. Level of monitoring specialisation (see Table 2 for definition)	No
18. Follow-up communication (see Table 2 for definition)	No
19. Integration (see Table 2 for definition)	No
20. Equity considerations (see Table 2 for definition)	No

Following the first round of definitions and sub-options for each category, up to 10 new articles were independently piloted for data extraction into a Macros-enabled Excel sheet by two research team members. The team then met to discuss uniformity and agreed on the approach for systematically mapping and extracting the rest of the articles. Using a macros-enabled workbook provided the functionality of using Visual Basic for Applications code- a programming language, to track our extracted data, build custom functions into the spreadsheet and configure the envisioned typology with automation features. Quality assurance processes were implemented such that extracted data from up to 40% of the articles were vetted by at least one other individual. During the extraction stage, we progressively excluded six information categories that were identified by group consensus as not relevant for standardizing RPM classification, as being ambiguous, or for being either too narrow or too wide in the range of options they encompassed, narrowing the number of categories from 20 to a final list of 12 (see Tables 1 and 2). The final list of categories intentionally makes no assumptions about scalability (i.e., ability to spread or support a particular number of patients served). Additionally, concepts pertaining to value and effectiveness were deemed out of scope due to a lack of empirical evidence to justify classification against these parameters. The draft version of the glossary outlining the 12 characteristics and completed extraction sheet were presented to the expert panel and other health system partners/RPM stakeholders in a meeting where the research team facilitated discussions on what a user-friendly tool for grouping RPM programs at a higher level, starting from the 12 identified characteristics and their associated subcategories could look like.

**Table 2:** Final list of 12 disease-agnostic characteristics of RPM programs.

Characteristic	Definition	Options
Alert Protocol	Refers to how a decision determines when escalation is needed where escalation can mean the RPM/clinical team contacts the patient, or the patient calls 911 or reaches out to other supportive services	Automatic Manual None Unknown
Data Entry Modality	Describes how physiologic measurements are collated/reported. *NOTE: this excludes patient-reported experience or outcome measures (PREMs/PROMs), symptoms, and patient surveys on experience as these are always manually entered.*	Fully automated Semi-automated Manual None Unknown
Data Access	Describes how measurements/collected data from the RPM program is streamlined or managed from the point of entry, where it is reviewed, or decisions are made	Centralized Fragmented Unknown
Manual Data Entry (Frequency)	Refers to the frequency for which the patient is required to manually enter data or report symptoms. *NOTE: This excludes automated measures.*	Daily Weekly Bi-Weekly Monthly None Unknown
Follow-up Communication	Describes how the RPM team stays connected or follows-up with the user (e.g., patient). Refers to general two-way communication between the clinical team and the patient. *NOTE: This excludes enrolment, onboarding, nor is it related to alerts.*	Synchronous on demand Asynchronous on demand Pre-scheduled None Unknown
Level of Monitoring Specialization	Describes the level of clinical specialization of the RPM team.	Moderately specialized No specialization Unknown
Availability of Team	Describes the availability of the RPM monitoring team.	24/7 Regular + weekends Regular workdays Irregular None Not applicable Unknown
Risk Profile	Refers to the severity of disease status for people enrolled into the RPM program.	Severe Moderate Low Non-specific Unknown
Integration Considerations	Refers to the extent to which the RPM program considered integration, including:1) with existing services and resources,2) with existing workflows, and3) with existing systems and infrastructure.	1 2 3 None Unknown
Vendor	Refers to the number of vendors in the RPM program including hardware and software devices (e.g., platform vendors, cloud services, digital applications, etc.)	Multiple integrated Multiple separate Single None Unknown
Device Ownership	Refers to the extent or burden on patient responsibility with respect to device ownership.	System provided Mixed ownership Bring Your Own Device (BYOD) Unknown
Equity Considerations	Refers to the extent to which the RPM program considered equity, including:1) language inclusivity (provides program in >1 language),2) digital literacy (provides regular support to users with little education or digital literacy),3) offline functionality (does not require users to have constant internet access and/or allows data to be collected offline and synced at a later time),4) culturally adapted (reports any considerations in making the RPM platform/program responsive to culture,5) digital access (provides all devices to the user and/or does not make the user possess a specific device or access to the internet as a pre-enrolment requirement), and6) access to one’s own personal health information (interface allows patients to see their own data).	3+ 1–2 None Unknown

## Results

Table 2 outlines the final list of 12 disease-agnostic categories and corresponding glossary.

In this section, we present the activities and outputs of Stage 3 of the KTA framework- i.e., the creation of knowledge tools and products.

### Defining and differentiating four domains of an RPM Typology

Building up from knowledge of implemented provincial RPM programs, expert feedback, and data from the extracted articles, and informed by project objectives, we clustered the 12 disease-agnostic characteristics of RPM programs into four domains, each defined below.

**Technology** - the level of automation and technological complexity of a given RPM program. It includes characteristics of manual data entry (e.g., patient reported outcome measures) and its frequency, the data entry modality (e.g., fully automated, semi-automated, manual for physiologic measurements), whether an alert protocol exists (e.g., triaging or flagging patients requiring follow-up from the clinical team), and data access (e.g., degree to which patients have access to their data).

**Touch** - the level of monitoring and interaction between patients and the clinical team. It includes characteristics related to the cadre of staff and level of clinical specialization of staff on the RPM team (e.g., less specialized nurse practitioners versus more specialized staff such as a cardiologist), how follow-up communication is facilitated, availability of the RPM team (e.g., 24/7 versus during usual business hours), and patients’ risk profile.

**Integration** - the extent to which the RPM program is linked to or leverages existing systems such as established clinical workflows, data interoperability, human resources, and vendor interconnectedness. It includes characteristics of device linkages (e.g., platform vendors, cloud services), and integration considerations (e.g., existing services and resources, workflows, systems, and infrastructure for patient records).

**Fig 2:**
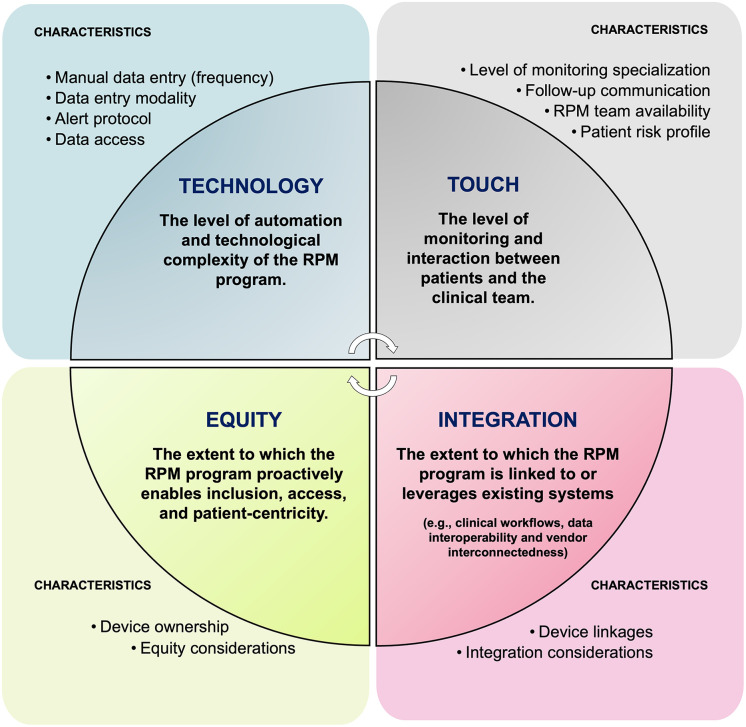
The four domains of the RPM Typology and their related characteristics. The 12 characteristics were defined through sense-making informed by two rapid literature reviews and feedback from an expert panel.

**Equity** - the extent to which the RPM program proactively enables inclusion, access, and patient-centricity. It includes the nature of device ownership and equity considerations (e.g., language, digital literacy, enabling offline functionality, cultural adaptations, provision of hardware/software and devices, data ownership and access). The domains are illustrated in Fig 2.

**Fig 3:**
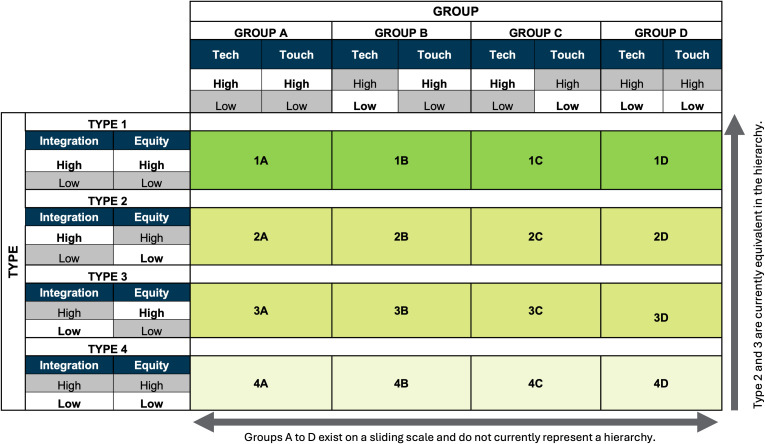
Overview of 16 distinct RPM typologies combining types and groups of four domains. A given RPM program can be mapped onto one of the 16 distinct typologies based on the program’s combination of characteristics. The greyed-out cells are to be ignored when reading the matrix.

To allow for differentiation within the four domains, we considered that each could be characterized as high or low. The domains **Technology** (called Tech for short) and **Touch** were called ***Groups*** and discussed as representative of expected variability of RPM program features with characteristics that exist on a sliding scale from low to high. The rationale for this is that the appropriate mix of technological complexity and monitoring intensity will likely be guided by pragmatic decisions related to budget, workforce availability, market competition with vendor options, program maturity, the population served, clinical pathway, available resources, etc. High-low combinations of these two domains were thus assumed to be equivalent (i.e., high tech is not considered to be superior to low tech RPM programs). We assigned alphabets to denote the four possible group combinations for Tech and Touch respectively - group A (high:high), group B (low:high), group C (high:low), and group D (low:low). See Fig 3.

In contrast, the other two domains - **Integration** and **Equity** (also known as patient-centricity), which together we called ***Types***, were agreed to represent inherent aspirations of all RPM programs. As such they were seen as program features that should be less prone to variation and more representative of a standard of aspiration. That is, all RPM programs should ideally be high integration and high equity. By following this approach in our distinction, we automatically created hierarchies for integration and equity – from low (Type 4) to high (Type 1). However, we assumed equivalence for mixed high and low combinations between Integration and Equity. That is, a high integration, low equity classification (Type 2) is equivalent to a low integration, high equity (Type 3) one. Finally, by combining the four *groups* with the four *types* we produced an alphanumeric categorization of RPM programs allowing users to categorize them as any of 16 distinct typologies (e.g., 1A, 4D, 2C, 3B, etc.). For example, a RPM program with typology 2B means it is characterized as low tech:high touch:high integration:low equity (see Fig 3). This can alternatively be written as ↓Tech ↑Touch ↑Intg ↓Eqty. If a RPM program is categorized as low tech:low touch:high integration:high equity (i.e., ↓ Tech ↓Touch ↑Intg ↑Eqty), it means it has a typology of 1D, and so on.

### Stage 3. Creation of knowledge products: Developing a survey and automated tool

#### Developing an RPM typology survey and automated typology categorization tool.

Using the defined typology characteristics from Stage 2, we created a typology survey (aka Typology Checklist) built into Microsoft Forms, designed to be completed electronically by program administrators (with the option to print out and complete a physical copy) and with questions that aligned with the information needed for Table 2. The Checklist can be completed within 10 minutes by those familiar with the RPM program in question to collect detailed semi-structured data about the features and characteristics needed to establish the categorization of the RPM program. The Typology Checklist contains 19 questions with multiple options to select from and includes an open-ended text box for additional comments and notes.

To map information systematically and reliably following completion of the Checklist by a RPM program administrator, we developed a detailed and user-friendly RPM typification tool, in Microsoft Excel, leveraging its developer feature which activates in-built advanced automation and interactive functionality. The final product automatically categorizes, in real time, any RPM program using a simple algorithm that responds to information about the program’s unique characteristics (Table 3).

**Table 3:** Snapshot of RPM Typology Tool.

	High (3)	Moderate (2)	Low (0–1)
**DOMAIN A: TECHNOLOGY**			
A1. Alert Protocol	[Automatic]	N/A	[Manual] or [None] or [Unknown]
A2. Data Entry Modality	[Fully-automated] or [None]	[Semi-automated]	[Manual] or [Unknown]
A3. Data Access	[Centralized]	N/A	[Fragmented] or [None] or [Unknown]
A4. Manual Data Entry (Frequency)	[Monthly] or [None]	[Weekly] or [Bi-weekly]	[Daily] or [Unknown]
**DOMAIN B: TOUCH**			
B1. Follow-up Communication	[Synchronous on demand]	[Asynchronous on demand]	[Pre-scheduled] or [None] or [Unknown]
B2. Level of Monitoring Specialization	[Moderately specialized]	N/A	[No specialization] or [None] or [Not applicable]
B3. Availability of Team	[24/7] or [Regular + weekends]	[Regular workdays]	[Irregular] or [None] or [Not applicable] or [Unknown]
B4. Risk Profile	[High]	[Moderate]	[Low] or [Non-specific] or [Unknown]
**DOMAIN C: INTEGRATION**			
C1. Integration Considerations*	[3] considerations	[2] considerations	[1] consideration or [None] or [Unknown]
C2. Device Linkages	[Multiple linked] or [Single]	N/A	[Multiple separate] or [None] or [Unknown]
**DOMAIN D: EQUITY/PATIENT-CENTRICITY**			
D1. Device Ownership	[System provided]	[Mixed ownership]	[BYOD]*** or [None] or [Unknown]
D2. Equity Considerations **	[5] or [6]	[3] or [4]	[1] or [2] or [None] or [Unknown]

*Integration considerations: Considers whether the RPM program is integrated with the following: (1) existing services and resources (i.e., shared full-time equivalents (FTE) with existing services and programs); (2) existing workflows (e.g., embedded into usual clinical visits and procedures such as intake processes), and; (3) existing systems and infrastructure (e.g., patient records, electronic medical records (EMRs) etc.) **Equity considerations: Considers whether the RPM program promotes equity by: (1) promoting language inclusivity (provides program in >1 language); (2) promoting digital literacy (provides regular support to users with little education or digital literacy); (3) enabling offline functionality (does not require users to have constant internet access and/or allows data to be collected offline and synced at a later time); (4) adapting the program culturally (reports any considerations in making the RPM platform/program responsive to culture); (5) providing patients digital access (provides all devices to the user and/or does not make the user possess a specific device or access to the internet as a pre-enrolment requirement), and; (6) enabling patients access to their own personal health information (interface allows patients to see their own data). *** BYOD: bring your own device

The algorithm consists of two interim calculations: one at the characteristic level (n = 12), and one at the domain level (n = 4). For the characteristic-level calculation, each characteristic is rated as being high (score: 3), moderate (score: 2), or low (score: 0 or 1). The domain-level calculation then computes the average across characteristic-level interim scores. When the domain-level score output is ≥ 2, that domain is automatically categorized as “high”, while domain-level scores < 2 are categorized as “low” for the respective domain. It is important to note that these interim calculations are used solely for the purposes of categorization and higher scores have no value implications- i.e., they do not imply “better” or “worse” RPM programs.

The full version of the typification tool is a multiple-tab Excel spreadsheet that includes a detailed user guide, typology glossary, user instructions, examples of six different RPM programs that have been typified and blank templates that could be duplicated and used to classify multiple RPM programs, with outputs visible in real time as selections of program features are made from pre-programmed drop-down functions in different cells. To download the RPM Typology Tool and Checklist please visit https://forms.gle/ofxSNKQMewo62uoj7

## Discussion

Using the RPM typology as a classification framework introduces a novel move from RPM as a illness- or clinical-centric model towards one that can support integrated disease-agnostic models of remote care. We identified four main domains that capture program complexity, integration, data flow and information management, and user needs. This classification approach can inform choice and design of RPM in ways that factor the individual, institutional, and resource needs of different clinical programs at varying stages of implementation maturity. Similar value and benefits have been reported in other health-oriented typologies [29].

Furthermore, using a standardized tool can facilitate benchmarking [32] and assist decision makers in comparing similar programs, identify which programs offer the most benefit for integrated and quality care, and determine areas that could benefit from additional investment or to enhance equity and patient-centricity. Our target end users for the RPM typology are health system administrators and policymakers who may use it to inform policy and to guide evaluation and investment decisions. Our intention is that the tool will also be valuable to researchers and implementers for planning and monitoring their own RPM projects as a companion to other implementation and evaluation strategies that are geared towards demonstrating robust program outcomes. For example, from the health system administrator and policymaker perspective, a type 2C program may have sufficient investment for integrating their program into existing services, workflows, and electronic medical records, but could benefit from additional investment into equity strategies to become classified as a type 1C program. Additionally, the appropriateness of intensity in each domain can be reevaluated over time considering program maturity, the population served, available resources, and plans for sustainability and scale. In a similar way researchers, implementers and those who design PRM programs can use this typology tool to inform design decisions and facilitate comparison in evaluations.

The ability to clearly identify the specific RPM typology (of 16 options) a program falls under can be used to assess its current position in relation to goals of integration and equity. It may also prompt consideration of alternative models - for example, determining whether resources allocated to high-tech approaches could be more effectively used in lower-tech models. Importantly, the intention of the RPM typology tool is not to replace established evaluation tools or frameworks. Rather, it is meant to be used in combination with existing evaluation tools which are crucial for understanding if RPM is working well (or not) and how it can be improved over time. Some examples include: implementation effectiveness evaluation tools such as the Proctor implementation outcome framework [33,34] or the consolidated framework for implementation research [35,36], multi-level assessment of digital health systems [37] or sources of complexity [38], and usability testing to incorporate patient user experiences [39].

While there is no one standard for designing RPM programs, integration and equity enhancing traits should be inherent baseline features of an optimal RPM program. This is reinforced through frameworks such as PROGRESS-PLUS [40], the digital determinants of health (DDoH) [40], and the health system framework for measuring health equity [32]. Furthermore, implementation teams should consider an appropriate mix of technology and touch against aspirational domains of integration and equity. For example, Type 1 may be considered aspirational with high scores on equity considerations and integration. However, the ideal typology (i.e., combination of Type and Group) may vary for each program based on factors including resources, program maturity, population served (clinical need and as a % of target group), as well as long-term goals for scale and sustainability.

### Benefit for health system payors- informing selection of technology vendors

Returning to the rationale for developing a disease-agnostic classification model for RPM, this typology can support evaluation of outcome data of RPM programs through contextualizing and characterizing different programs similarly. The problem our study aimed to solve is the current inability to compare between or across disease-focused programs due to high variability resulting from a solely clinical focus for describing RPM programs. Using this novel typology addresses this problem- four programs that are similarly classified as 4C can be reasonably compared to another group of programs that are similarly classified as 1D, even when each program serves different clinical groups or socio demographic populations.

This characterization allows for clustering of different types of RPM programs to compare “apples to apples” given the high variability and variety of different types of RPM programs within and across disease domains. A shortlist of vendors of record can assist organizations and health teams with selecting an approved and verified vendor to suit their program needs. For example, type 2A and 2B RPM programs vary only by the level of technology. If effectiveness evidence is compared and the low-tech options have comparable effectiveness, this could considerably reduce the cost of adopting a similar program (either within the same clinical domain or applied to a different one). Similarly, if a hypothetical new RPM program is in the planning and preparation stage, comparing effectiveness across 1A and 1C typologies, which differ only by high vs low touch, can inform optimised approaches to balance cost and time with anticipated effectiveness.

### Future directions and limitations

The RPM typology as a tool to classify diverse RPM programs is primarily based on evidence from an environmental scan of international programs in the chronic disease and COVID-19 domains at various stages of implementation (e.g., pre-pilot, pilot, fully integrated, etc.). Sensemaking that informed which characteristics and domains were prioritized was thus influenced by the limits of information provided within journal articles. It is possible to include additional domains of interest that were not captured in the literature but might be relevant to decision-makers and innovators. For example, although we recognized its importance, scalability, such as the spread (number of sites) and caseload (number of patients served), was not sufficiently captured in the literature and thus is not included in the current version of the typology. Similarly, the current tool did not consider program maturity or implementation context, which could inform additional layers of granularity to how each typology category is understood. The aspiration of high integration and high equity models may therefore not necessarily be optimal depending on the context and environment. Notably the literature knowledge base which informed the typology focused on OECD countries, resulting in underrepresentation of low- and middle-income countries. The implications of these limitations are linked to critiques of knowledge to action models like the KTA, wherein community and non-formalised knowledge are deprioritised and there is limited utility in engaging with social justice and equity [41]. Furthermore, the current typology has not yet been validated and the scoring system used assumes equal weighting for all characteristics. The immediate next step is to conduct validity testing of this tool. A future cluster analysis would be beneficial for comparing efficacy across programs with different clinical pathways to further inform baseline features for different typologies and to validate the assumption regarding integration and equity features existing on a hierarchy from low (Type 4) to high (Type 1).

## Conclusion

With an increasing burden of multimorbidity, remote care could enhance both access and coordination of care closer to home or in the community. Disease-agnostic RPM platforms can allow for more efficient patient-centered care, focusing on integrated delivery that considers the appropriate level of technological complexity and monitoring intensity. This requires a shift in the current approach to the design and implementation of RPM programs that our novel typology addresses. By applying this classification framework to support standardized reporting and systematic comparison of RPM programs, we hope that new investments will have greater potential to demonstrate how they can be best positioned to deliver cost-effective and efficient care. Technology vendors and implementing organisations can also be better informed on how to approach the intersection of technological and systems integration with equitable access in their respective RPM programs.

## Supporting information

S1 Table Provides an overview of search strategies for both Embase and Medline for the rapid literature reviews. (DOCX)
