# Supplementary material for: Design and development of a disease-agnostic remote patient monitoring typology and associated tools
Source: PLOS Digit Health. 2026 Jul 31;5(7):e0001595. doi: 10.1371/journal.pdig.0001595 (PMC13426941; doi:10.1371/journal.pdig.0001595)
Supplement: S1 Table — (DOCX) [file pdig.0001595.s001.docx]

Supporting Information 1 – Search Strategies

This document contains the Embase and Medline Search Strategies

## Embase Search:

| **Embase Classic+Embase <1947 to 2022 March 24>**   \| **#** \| **Searches** \| **Results** \| **Type** \| \| --- \| --- \| --- \| --- \| \| 1 \| teleconsultation/ or electronic consultation/ or telemedicine/ or telecardiology/ or teleconsultation/ or telediagnosis/ or telemonitoring/ or telepathology/ or telepharmacy/ or video consultation/ or remote sensing/ \| 63816 \| Advanced \| \| 2 \| ((computer or distance or internet or phone or online or remote or tele* or video or virtual or web) adj2 (administ* or advice or assess* or care or chat* or confer* or consult* or counsel* or deliver* or health* or interv* or manag* or medic* or monitor or nurs* or pharm* or therap* or visit*)).ti,ab,kf. \| 138572 \| Advanced \| \| 3 \| (remot* adj4 monitor*).tw,kf. \| 9088 \| Advanced \| \| 4 \| (teleadminist* or teleassess* or telecare or telechat* or teleconf* or teleconsult* or teledeliv* or telehealth* or teleinterv* or telemanag* or telemedic* or telemonit* or telenurs* or telepharm* or televisit* or teletherap* or videochat* or videotelephon* or videophone* or wireless tech* or telecardiology or telehypertension or telepathology or smart device or smart phone).tw,kf. \| 47476 \| Advanced \| \| 5 \| (eConsult* or e-consult* or eHealth* or e-Health* or einterv* or e-interv* or etherap* or e-therap* or mHealth* or m-Health* or mobile health* or Mobile application*).tw,kf. \| 27079 \| Advanced \| \| 6 \| Telemed*.jw. \| 7281 \| Advanced \| \| 7 \| mobile application/ or mobile health application/ \| 19257 \| Advanced \| \| 8 \| (App or apps or facetime* or skype* or zoom or webbased tool or web-based tool* or voice-over or voiceover or VoIP).tw,kf. \| 58617 \| Advanced \| \| 9 \| or/1-8 [remote monitoring] \| 272295 \| Advanced \| \| 10 \| obstructive lung disease/ or chronic obstructive lung disease/ \| 155689 \| Advanced \| \| 11 \| (aecb or chronic airflow disease* or chronic airflow disorder* or chronic airflow limitation* or chronic airway disease* or chronic airway disorder* or chronic airway limitation* or chronic obstructive airflow disease* or chronic obstructive airway disease* or chronic obstructive airway disorder* or coad or cobd or copd or emphysema*).tw,kf. \| 142143 \| Advanced \| \| 12 \| ((chronic* or persistent) adj3 bronchiti*).tw,kf. \| 18655 \| Advanced \| \| 13 \| (obstruct* adj3 (pulmonary or lung* or airway* or airflow* or bronch* or respirat*)).tw,kf. \| 148721 \| Advanced \| \| 14 \| or/10-13 [COPD] \| 278454 \| Advanced \| \| 15 \| diabetes mellitus/ or exp diabetic complication/ or impaired glucose tolerance/ or insulin dependent diabetes mellitus/ or lipoatrophic diabetes mellitus/ or non insulin dependent diabetes mellitus/ \| 1092809 \| Advanced \| \| 16 \| diabetic ketoacidosis/ \| 15767 \| Advanced \| \| 17 \| (diabet* or NIDDM or IDDM or prediabet* or MODY or T1DM or T2DM or T1D or T2D or non insulin* depend* or noninsulin* depend* or noninsulindepend* or non insulin?depend*).tw,kf. \| 1120429 \| Advanced \| \| 18 \| or/15-17 [diabetes] \| 1340028 \| Advanced \| \| 19 \| exp heart failure/ \| 602725 \| Advanced \| \| 20 \| (decompensation cordis or myocardial decompensation or chronic heart failure or chronic cardiac failure).tw,kf. \| 31133 \| Advanced \| \| 21 \| ((Heart or cardiac or myocardial) adj2 (failure or chronic or decompensation or congestive)).tw,kf. \| 368855 \| Advanced \| \| 22 \| ((left ventricular or left ventricle) adj2 (failure or insufficien* or dysfunction*)).tw,kf. \| 38521 \| Advanced \| \| 23 \| ((dilated or congestive) adj2 cardiomyopath*).tw,kf. \| 31958 \| Advanced \| \| 24 \| ((ventricular or ventricle*) adj2 (failure or insufficien* or dysfunction*)).tw,kf. \| 57105 \| Advanced \| \| 25 \| lvsd.tw,kf. \| 1452 \| Advanced \| \| 26 \| or/19-25 [heart failure] \| 702166 \| Advanced \| \| 27 \| hypertension/ or elevated blood pressure/ or borderline hypertension/ or essential hypertension/ or hereditary hypertension/ or hypertensive crisis/ or malignant hypertension/ or masked hypertension/ or orthostatic hypertension/ or prehypertension/ or renovascular hypertension/ or resistant hypertension/ or systolic hypertension/ \| 756479 \| Advanced \| \| 28 \| exp blood pressure/ \| 691810 \| Advanced \| \| 29 \| (hypertens* or prehypertens*).tw,kf. \| 779522 \| Advanced \| \| 30 \| ((blood or arterial or diastolic or systolic) adj3 pressure).tw,kf. \| 600352 \| Advanced \| \| 31 \| ((elevat* or increas* or lower or high or rais* or rising) adj2 (bp or dbp or hbp or sbp)).tw,kf. \| 26253 \| Advanced \| \| 32 \| or/27-31 [hypertension] \| 1581351 \| Advanced \| \| 33 \| COVID-19/ or exp COVID-19 Testing/ or COVID-19 Vaccines/ or SARS-CoV-2/ \| 73092 \| Advanced \| \| 34 \| (coronavirus/ or betacoronavirus/ or coronavirus infections/) and (disease outbreaks/ or epidemics/ or pandemics/) \| 10386 \| Advanced \| \| 35 \| (nCoV* or 2019nCoV or 19nCoV or COVID19* or COVID or SARS-COV-2 or SARSCOV-2 or SARS-COV2 or SARSCOV2 or SARS coronavirus 2 or Severe Acute Respiratory Syndrome Coronavirus 2 or Severe Acute Respiratory Syndrome Corona Virus 2).tw,kf. \| 241539 \| Advanced \| \| 36 \| ((new or novel or "2019" or Wuhan or Hubei or China or Chinese) adj3 (coronavirus* or corona virus* or betacoronavirus* or CoV or HCoV)).ti,ab,kf,ot. \| 55596 \| Advanced \| \| 37 \| (longCOVID* or postCOVID* or postcoronavirus* or postSARS*).ti,ab,kf,ot. \| 81 \| Advanced \| \| 38 \| ((coronavirus* or corona virus* or betacoronavirus*) adj3 (pandemic* or epidemic* or outbreak* or crisis)).ti,ab,kf,ot. \| 11478 \| Advanced \| \| 39 \| ((Wuhan or Hubei) adj5 pneumonia).ti,ab,kf,ot. \| 451 \| Advanced \| \| 40 \| or/33-39 \| 252183 \| Advanced \| \| 41 \| limit 40 to yr=2019 -Current [COVID] \| 250746 \| Advanced \| \| 42 \| 14 or 18 or 26 or 32 or 41 \| 3500422 \| Advanced \| \| 43 \| 9 and 42 \| 47477 \| Advanced \| \| 44 \| limit 43 to (english and last 5 years) \| 33192 \| Advanced \| |
| --- | --- | --- | --- | --- | --- | --- | --- | --- | --- | --- | --- | --- | --- | --- | --- | --- | --- | --- | --- | --- | --- | --- | --- | --- | --- | --- | --- | --- | --- | --- | --- | --- | --- | --- | --- | --- | --- | --- | --- | --- | --- | --- | --- | --- | --- | --- | --- | --- | --- | --- | --- | --- | --- | --- | --- | --- | --- | --- | --- | --- | --- | --- | --- | --- | --- | --- | --- | --- | --- | --- | --- | --- | --- | --- | --- | --- | --- | --- | --- | --- | --- | --- | --- | --- | --- | --- | --- | --- | --- | --- | --- | --- | --- | --- | --- | --- | --- | --- | --- | --- | --- | --- | --- | --- | --- | --- | --- | --- | --- | --- | --- | --- | --- | --- | --- | --- | --- | --- | --- | --- | --- | --- | --- | --- | --- | --- | --- | --- | --- | --- | --- | --- | --- | --- | --- | --- | --- | --- | --- | --- | --- | --- | --- | --- | --- | --- | --- | --- | --- | --- | --- | --- | --- | --- | --- | --- | --- | --- | --- | --- | --- | --- | --- | --- | --- | --- | --- | --- | --- | --- | --- | --- | --- | --- | --- | --- | --- | --- | --- | --- |

## Medline Search:

| **Ovid MEDLINE: Epub Ahead of Print, In-Process & Other Non-Indexed Citations, Ovid MEDLINE® Daily and Ovid MEDLINE® <1946-Present>**   \| **#** \| **Searches** \| **Results** \| **Type** \| \| --- \| --- \| --- \| --- \| \| 1 \| Remote Consultation/ or exp Telemedicine/ or Remote Sensing Technology/ \| 42883 \| Advanced \| \| 2 \| ((computer or distance or internet or phone or online or remote or tele* or video or virtual or web) adj2 (administ* or advice or assess* or care or chat* or confer* or consult* or counsel* or deliver* or health* or interv* or manag* or medic* or monitor or nurs* or pharm* or therap* or visit*)).ti,ab,kf. \| 99800 \| Advanced \| \| 3 \| (remot* adj4 monitor*).tw,kf. \| 5864 \| Advanced \| \| 4 \| (teleadminist* or teleassess* or telecare or telechat* or teleconf* or teleconsult* or teledeliv* or telehealth* or teleinterv* or telemanag* or telemedic* or telemonit* or telenurs* or telepharm* or televisit* or teletherap* or videochat* or videotelephon* or videoconsultation or videophone* or wireless tech* or telecardiology or telehypertension or smart device or smart phone or electronic consultation or telediagnosis or telepathology).ti,ab,kf. \| 34882 \| Advanced \| \| 5 \| (eConsult* or e-consult* or eHealth* or e-Health* or einterv* or e-interv* or etherap* or e-therap* or mHealth* or m-Health* or mobile health* or Mobile application*).ti,ab,kf. \| 24301 \| Advanced \| \| 6 \| Telemed*.jw. \| 6562 \| Advanced \| \| 7 \| exp Mobile Applications/ \| 9689 \| Advanced \| \| 8 \| (App or apps or facetime* or skype* or zoom or webbased tool or web-based tool* or voice-over or voiceover or VoIP).ti,ab,kf. \| 42275 \| Advanced \| \| 9 \| or/1-8 [Remote monitoring] \| 194481 \| Advanced \| \| 10 \| exp lung diseases, obstructive/ \| 226599 \| Advanced \| \| 11 \| (aecb or chronic airflow disease* or chronic airflow disorder* or chronic airflow limitation* or chronic airway disease* or chronic airway disorder* or chronic airway limitation* or chronic obstructive airflow disease* or chronic obstructive airway disease* or chronic obstructive airway disorder* or coad or cobd or copd or emphysema*).tw,kf. \| 80946 \| Advanced \| \| 12 \| ((chronic* or persistent) adj3 bronchiti*).tw,kf. \| 11587 \| Advanced \| \| 13 \| (obstruct* adj3 (pulmonary or lung* or airway* or airflow* or bronch* or respirat*)).tw,kf. \| 97872 \| Advanced \| \| 14 \| or/10-13 [COPD] \| 294419 \| Advanced \| \| 15 \| exp Diabetes Mellitus, Type 2/ or Diabetes, Gestational/ or exp Diabetes Complications/ or Diabetes Mellitus/ or Diabetes Insipidus/ or Diabetes Mellitus, Type 1/ \| 442769 \| Advanced \| \| 16 \| Diabetic Ketoacidosis/ \| 7056 \| Advanced \| \| 17 \| (diabet* or NIDDM or IDDM or prediabet* or MODY or T1DM or T2DM or T1D or T2D or non insulin* depend* or noninsulin* depend* or noninsulindepend* or non insulin?depend*).tw,kf. \| 718962 \| Advanced \| \| 18 \| or/15-17 [diabetes] \| 775233 \| Advanced \| \| 19 \| exp Heart Failure/ \| 136166 \| Advanced \| \| 20 \| (decompensation cordis or myocardial decompensation or chronic heart failure or chronic cardiac failure).tw,kf. \| 18001 \| Advanced \| \| 21 \| ((Heart or cardiac or myocardial) adj2 (failure or chronic or decompensation or congestive)).tw,kf. \| 218071 \| Advanced \| \| 22 \| ((left ventricular or left ventricle) adj2 (failure or insufficien* or dysfunction*)).tw,kf. \| 23536 \| Advanced \| \| 23 \| ((dilated or congestive) adj2 cardiomyopath*).tw,kf. \| 20122 \| Advanced \| \| 24 \| ((ventricular or ventricle*) adj2 (failure or insufficien* or dysfunction*)).tw,kf. \| 33857 \| Advanced \| \| 25 \| lvsd.tw,kf. \| 611 \| Advanced \| \| 26 \| or/19-25 [heart failure] \| 281251 \| Advanced \| \| 27 \| exp Hypertension/ or Blood Pressure/ elevated blood pressure/ \| 509349 \| Advanced \| \| 28 \| (hypertens* or prehypertens*).tw,kf. \| 480942 \| Advanced \| \| 29 \| ((blood or arterial or diastolic or systolic) adj3 pressure).tw,kf. \| 401371 \| Advanced \| \| 30 \| ((elevat$ or increas$ or lower or high or rais$ or rising) adj2 (bp or dbp or hbp or sbp)).tw,kf. \| 15907 \| Advanced \| \| 31 \| or/27-30 [hyper tension] \| 878249 \| Advanced \| \| 32 \| COVID-19/ or exp COVID-19 Testing/ or COVID-19 Vaccines/ or SARS-CoV-2/ \| 149778 \| Advanced \| \| 33 \| (coronavirus/ or betacoronavirus/ or coronavirus infections/) and (disease outbreaks/ or epidemics/ or pandemics/) \| 40110 \| Advanced \| \| 34 \| (nCoV* or 2019nCoV or 19nCoV or COVID19* or COVID or SARS-COV-2 or SARSCOV-2 or SARS-COV2 or SARSCOV2 or SARS coronavirus 2 or Severe Acute Respiratory Syndrome Coronavirus 2 or Severe Acute Respiratory Syndrome Corona Virus 2).ti,ab,kf,nm,ot,ox,rx,px. \| 229256 \| Advanced \| \| 35 \| ((new or novel or "2019" or Wuhan or Hubei or China or Chinese) adj3 (coronavirus* or corona virus* or betacoronavirus* or CoV or HCoV)).ti,ab,kf,ot. \| 56136 \| Advanced \| \| 36 \| (longCOVID* or postCOVID* or postcoronavirus* or postSARS*).ti,ab,kf,ot. \| 31 \| Advanced \| \| 37 \| ((coronavirus* or corona virus* or betacoronavirus*) adj3 (pandemic* or epidemic* or outbreak* or crisis)).ti,ab,kf,ot. \| 11732 \| Advanced \| \| 38 \| ((Wuhan or Hubei) adj5 pneumonia).ti,ab,kf,ot. \| 387 \| Advanced \| \| 39 \| or/32-38 \| 240063 \| Advanced \| \| 40 \| limit 39 to yr=2019 -Current [COVID] \| 238595 \| Advanced \| \| 41 \| 14 or 18 or 26 or 31 or 40 \| 2220236 \| Advanced \| \| 42 \| 9 and 41 \| 30977 \| Advanced \| \| 43 \| Animals/ not (Animals/ and Humans/) \| 4943573 \| Advanced \| \| 44 \| 42 not 43 \| 30641 \| Advanced \| \| 45 \| limit 44 to (english and last 5 years) \| 22533 \| Advanced \| |
| --- | --- | --- | --- | --- | --- | --- | --- | --- | --- | --- | --- | --- | --- | --- | --- | --- | --- | --- | --- | --- | --- | --- | --- | --- | --- | --- | --- | --- | --- | --- | --- | --- | --- | --- | --- | --- | --- | --- | --- | --- | --- | --- | --- | --- | --- | --- | --- | --- | --- | --- | --- | --- | --- | --- | --- | --- | --- | --- | --- | --- | --- | --- | --- | --- | --- | --- | --- | --- | --- | --- | --- | --- | --- | --- | --- | --- | --- | --- | --- | --- | --- | --- | --- | --- | --- | --- | --- | --- | --- | --- | --- | --- | --- | --- | --- | --- | --- | --- | --- | --- | --- | --- | --- | --- | --- | --- | --- | --- | --- | --- | --- | --- | --- | --- | --- | --- | --- | --- | --- | --- | --- | --- | --- | --- | --- | --- | --- | --- | --- | --- | --- | --- | --- | --- | --- | --- | --- | --- | --- | --- | --- | --- | --- | --- | --- | --- | --- | --- | --- | --- | --- | --- | --- | --- | --- | --- | --- | --- | --- | --- | --- | --- | --- | --- | --- | --- | --- | --- | --- | --- | --- | --- | --- | --- | --- | --- | --- | --- | --- | --- | --- | --- | --- | --- |
