# Supplementary material for: Design and development of a disease-agnostic remote patient monitoring typology and associated tools
Source: PLOS Digit Health. 2026 Jul 31;5(7):e0001595. doi: 10.1371/journal.pdig.0001595 (PMC13426941; doi:10.1371/journal.pdig.0001595)
Supplement: Attachment — Submitted filename: Response to Reviewers_08MAY2026.docx [file pdig.0001595.s003.docx]

**Reviewer Comments and Author’s Point-by-Point Responses**

**Reviewer’s General Comment:** The manuscript addresses an important gap in remote patient monitoring currently, specifically the lack of a standardized framework for classifying diverse RPM programs. The rationale is well articulated, and the authors’ Knowledge to Action methodology is logical and transparent. The proposed typology (Technology, Touch, Integration, and Equity) is a meaningful contribution with hopefully potential use in evaluation and program design. The manuscript is generally well written, however, some areas could be iterated to aid clarity, methodological justification, and the tool’s validity and limitations. Particular attention should be paid to formatting long sentences with multiple points, specifically in the latter sections, to assist with flow and readability.

**General Response:** We thank the reviewer for the time and care taken to review our manuscript, and for the detailed and constructive comments provided. We appreciate the opportunity to strengthen the manuscript and make general revisions to enhance readability and flow. In the point-by-point response below, the line numbers referenced are based on the clean version of the revised manuscript.

**Abstract and Author summary**

Line 28: either expand why a macros enabled Excel is useful in this context, or perhaps a structured description in the appendices to reinforce the scoring algorithm for reproducibility.

**Response:** Thank you for this suggestion. To stay within the word limit for the abstract, we have provided additional information on why a macros-enabled template was used in the main body of the manuscript (lines 214 – 217) within the methods section: *Using a macros-enabled workbook provided the functionality of using Visual Basic for Applications code- a programming language, to track our extracted data, build custom functions into the spreadsheet and configure the envisioned typology with automation features.”*

**Introduction**

Line 90-93: Tying this back into chronic conditions above (Line 84-87) would serve the important point that equity can be eroded further in long-term conditions.

Line 102-103: It may be helpful to note further why this typology (and the studies used therein) are providing a novel/homogenous framework as one would assume that the 40 RPM programs under review by the MOH and OH have similar threads or themes. This is touched on (Line 105-106), but it be expanded to make it explicit for the reader.

**Response:** We have revised the latter paragraphs of the introductory section (lines 105 – 111) to emphasize the novelty and rationale for developing a framework that strengthens evaluation and decision-making evidence regarding RPM.

**Methods**

- Line 145-138: Justifying why these four conditions and restricting to 2021 would be needed here.

- Line 158: Same as above, you need to justify why the search was restricted to 2022.

- Line 164-166: Why 110? A justification would be appropriate here.

- Line 169-172: The OECD point is valid, but you should discuss the trade-offs. Excluding the non-OECD countries may omit innovative or impactful RPM studies, especially in LMIC contexts where RPM adoption is growing.

- Line 180-185, 218-224: This is quite repetitive and hard to track, it would be easier for the reader to reference one table that shows the initial characteristics, the next column that shows those removed, and the final column with the final list. You can build a structure/narrative around this table that explains the process much easier.

**Response:** We appreciate the feedback and have made the following revisions:

**-** Justification has been provided in line 150 for the choice of the four chronic conditions (*“…- conditions where RPM has been most widely used.”)* and the date limit in lines 142-143 which has been corrected to 2022 (*“Next, we carried out two rapid literature reviews in 2022 – one focusing on RPM programs for chronic diseases, and the other on COVID-19….”*). 2022 was the year in which the searches were run, hence the end date.

**-** The rationale for setting a target of 110 articles in already provided in lines 167 – 170: *“Our intention was to select approximately 110 articles (20 publications per disease category- four chronic conditions and COVID-19, with an additional 10 for multi-disease RPM programs) from the 218 total identified, using non-probability quota sampling, a strategy to ensure diversity and relevance when managing large bodies of evidence.”* Given the aim of the review- to characterise the ways in which RPM programs have been configured and inform a standard for classifyijng different programs, it was neither practical nor necessary to extract data from all available articles on RPM programs.

**-** In the revised limitations section, we now acknowledge the trade-offs in choice to focus on OECD countries (lines 428 - 430): *“Notably the literature knowledge base which informed the typology focused on OECD countries, resulting in underrepresentation of low- and middle-income countries.”*

**-** A new Table 1 has been included in the revised article outlining the initial list of 20 categories and those that did not make it to the final list (lines 207 – 209). The final list was already captured in the original manuscript- formerly Table 1, now Table 2 (lines 232 – 234). These revisions have further enhanced readability and minimised a lengthy narrative.

**Results**

- Table 1: Providing the Options column as a vertical list would be helpful for the reader.

- Line 244-301: This section is rightly in depth and very detailed, however the structure could be reconsidered to help the reader build upon the relevant information into the figures provided. For example, introduce the four domains and their descriptions (Line 253-272) first, then how these are summarised with characteristics (Figure 1), which then flows into the differentiation of the four domains (Line 273-283).

- Line 282-288: Providing examples of actual typology combinations would help the readers’ interpretability.

- Line 308-312: As mentioned previously, the formatting and grammar from this point would be worth reviewing. For example, multiple unnecessary clauses: “The Checklist, which can be completed in 5 to 10 minutes by an individual very familiar with the RPM program in question was to be used to collect sufficiently detailed semi-structured data about any given RPM program’s features and characteristics such that the information could then be used to establish the categorization of the RPM program.” This could be: “The Checklist can be completed within 10 minutes by those familiar with the RPM program in question to collect detailed semi-structured data about the features and characteristics to establish the categorization of the RPM program”.

- Line 316: Similar to the macros comment above, either expand why the developer features were used or provide a clear description in the appendices so the reader is aware of the process.

**Response:** Thank you for the feedback.

- Table 2 has been revised such that the choices in column 3 (Options) are listed vertically. See (lines 232 – 234).

- We have revised the sequence of content to enhance readability and flow as suggested by the reviewer

- We have included additional sentences (see lines 295 – 299) with two examples of typology combinations to explain how figure 2 should be interpreted: *“For example, a RPM program with typology 2B means it is characterized as low tech:high touch:high integration:low equity (see Fig 2). This can alternatively be written as ↓Tech ↑Touch ↑Intg ↓Eqty. If a RPM program is categorized as low tech:low touch:high integration:high equity (i.e. ↓Tech ↓Touch ↑Intg ↑Eqty), it means it has a typology of 1D, and so on.”*

- Former lines 308-312 (now lines 306 – 309) have been revised and now read: *“The Checklist can be completed within 10 minutes by those familiar with the RPM program in question to collect detailed semi-structured data about the features and characteristics needed to establish the categorization of the RPM program.”*

*-* The developer feature in Excel is a technical feature of Excel that enables access to advanced automation tools and ability to design interactive user forms, which basic users might not be aware of. This is a technical skill (similar to how certain functionalities in R software are known and familiar to more advanced users of the program) and beyond the scope of the paper to detail for readers. As with the previous comment on macros, we have extended the sentence to explain briefly (lines 312 – 314): *“…we developed a detailed and user-friendly RPM typification tool, in Microsoft Excel, leveraging its developer feature which activates in-built advanced automation and interactive functionality in Excel.”*

**Discussion**

- Line 418-: The limitations are duly noted, however, detailing how community-based or LMIC programs may be underrepresented is key here. Tied to this, it may be fair to acknowledge that high-equity and high-integration models may not always be optimal depending on the context and environment.

- Tying in a critique of the KTA model would also be appropriate in the limitations, for example, marginalising community expertise and resource and its usefulness in addressing structural inequities, which provides an opportunity to reflect on your approach using this in the present study.

**Response:** Thank you for this suggestion to critically reflect on and acknowledge the limitations that are inherent in and inadvertently compound through the selection criteria of the literature review as well as the KTA framework. We agree that given contextual variations and in line with our earlier stated point that the categorisations do not imply a better or worse judgement on RPM programs, we should explicitly note that the aspirational domains of integration and equity may not necessarily be optimal. This revision is now captured in lines 426 – 432: *“The aspiration of high integration and high equity models may therefore not necessarily be optimal depending on the context and environment. Notably the literature knowledge base which informed the typology focused on OECD countries, resulting in underrepresentation of low- and middle-income countries. The implications of these limitations are linked to critiques of knowledge to action models like the KTA, wherein community and non-formalised knowledge are deprioritised and there is limited utility in engaging with social justice and equity (42).”*

**General**

- Formatting needs some attention, for example, page numbers start on pg10, and Figure 3 is the first to appear on pg9.

**Response:** Apologies for this error; there should have been no page numbers in the submission file and this has been removed in the revised submission.
