# Supplementary material for: Design and development of a disease-agnostic remote patient monitoring typology and associated tools
Source: PLOS Digit Health. 2026 Jul 31;5(7):e0001595. doi: 10.1371/journal.pdig.0001595 (PMC13426941; doi:10.1371/journal.pdig.0001595)
Supplement: Peer review history

# Peer review: Decision Letter 0

**DOI:** 10.1371/journal.pdig.0001595.r001

**Authors:** Hongxing Luo, Shuduo Zhou

24 Feb 2026

PDIG-D-25-00742

Design and development of a disease-agnostic remote patient monitoring typology and associated tools

PLOS Digital Health

Dear Dr. Abejirinde,

Thank you for submitting your manuscript to PLOS Digital Health. After careful consideration, we feel that it has merit but does not fully meet PLOS Digital Health's publication criteria as it currently stands. Therefore, we invite you to submit a revised version of the manuscript that addresses the points raised during the review process.

Please submit your revised manuscript by Apr 25 2026 11:59PM. If you will need more time than this to complete your revisions, please reply to this message or contact the journal office at digitalhealth@plos.org. When you're ready to submit your revision, log on to https://www.editorialmanager.com/pdig/ and select the 'Submissions Needing Revision' folder to locate your manuscript file.

Please include the following items when submitting your revised manuscript:

* A letter that responds to each point raised by the editor and reviewer(s). You should upload this letter as a separate file labeled 'Response to Reviewers'. This file does not need to include responses to any formatting updates and technical items listed in the 'Journal Requirements' section below.

* A marked-up copy of your manuscript that highlights changes made to the original version. You should upload this as a separate file labeled 'Revised Manuscript with Track Changes'.

* An unmarked version of your revised paper without tracked changes. You should upload this as a separate file labeled 'Manuscript'.

If you would like to make changes to your financial disclosure, competing interests statement, or data availability statement, please make these updates within the submission form at the time of resubmission. Guidelines for resubmitting your figure files are available below the reviewer comments at the end of this letter.

We look forward to receiving your revised manuscript.

Kind regards,

Shuduo Zhou

Hongxing Luo

Leo Anthony Celi

PLOS Digital Health

orcid.org/0000-0001-6712-6626

**Journal Requirements:**

If the reviewer comments include a recommendation to cite specific previously published works, please review and evaluate these publications to determine whether they are relevant and should be cited. There is no requirement to cite these works unless the editor has indicated otherwise.

**Additional Editor Comments (if provided):**

[Note: HTML markup is below. Please do not edit.]

**Reviewers' Comments:**

Reviewer's Responses to Questions

**Comments to the Author**

1. Does this manuscript meet PLOS Digital Health’s publication criteria? Is the manuscript technically sound, and do the data support the conclusions? The manuscript must describe methodologically and ethically rigorous research with conclusions that are appropriately drawn based on the data presented.

Reviewer #1: Yes

**********

2. Has the statistical analysis been performed appropriately and rigorously?

Reviewer #1: N/A

**********

3. Have the authors made all data underlying the findings in their manuscript fully available (please refer to the Data Availability Statement at the start of the manuscript PDF file)?

The PLOS Data policy requires authors to make all data underlying the findings described in their manuscript fully available without restriction, with rare exception. The data should be provided as part of the manuscript or its supporting information, or deposited to a public repository. For example, in addition to summary statistics, the data points behind means, medians and variance measures should be available. If there are restrictions on publicly sharing data—e.g. participant privacy or use of data from a third party—those must be specified.

Reviewer #1: Yes

**********

4. Is the manuscript presented in an intelligible fashion and written in standard English?

PLOS Digital Health does not copyedit accepted manuscripts, so the language in submitted articles must be clear, correct, and unambiguous. Any typographical or grammatical errors should be corrected at revision, so please note any specific errors here.

Reviewer #1: Yes

**********

5. Review Comments to the Author

Please use the space provided to explain your answers to the questions above. You may also include additional comments for the author, including concerns about dual publication, research ethics, or publication ethics. (Please upload your review as an attachment if it exceeds 20,000 characters)

Reviewer #1: The manuscript addresses an important gap in remote patient monitoring currently, specifically the lack of a standardized framework for classifying diverse RPM programs. The rationale is well articulated, and the authors’ Knowledge to Action methodology is logical and transparent.

The proposed typology (Technology, Touch, Integration, and Equity) is a meaningful contribution with hopefully potential use in evaluation and program design.

The manuscript is generally well written, however, some areas could be iterated to aid clarity, methodological justification, and the tool’s validity and limitations. Particular attention should be paid to formatting long sentences with multiple points, specifically in the latter sections, to assist with flow and readability.

Feedback:

Abstract and Author summary

Line 28: either expand why a macros enabled Excel is useful in this context, or perhaps a structured description in the appendices to reinforce the scoring algorithm for reproducibility.

Introduction

Line 90-93: Tying this back into chronic conditions above (Line 84-87) would serve the important point that equity can be eroded further in long-term conditions.

Line 102-103: It may be helpful to note further why this typology (and the studies used therein) are providing a novel/homogenous framework as one would assume that the 40 RPM programs under review by the MOH and OH have similar threads or themes. This is touched on (Line 105-106), but it be expanded to make it explicit for the reader.

Methods

Line 145-138: Justifying why these four conditions and restricting to 2021 would be needed here.

Line 158: Same as above, you need to justify why the search was restricted to 2022.

Line 164-166: Why 110? A justification would be appropriate here.

Line 169-172: The OECD point is valid, but you should discuss the trade-offs. Excluding the non-OECD countries may omit innovative or impactful RPM studies, especially in LMIC contexts where RPM adoption is growing.

Line 180-185, 218-224: This is quite repetitive and hard to track, it would be easier for the reader to reference one table that shows the initial characteristics, the next column that shows those removed, and the final column with the final list. You can build a structure/narrative around this table that explains the process much easier.

Results

Table 1: Providing the Options column as a vertical list would be helpful for the reader.

Line 244-301: This section is rightly in depth and very detailed, however the structure could be reconsidered to help the reader build upon the relevant information into the figures provided. For example, introduce the four domains and their descriptions (Line 253-272) first, then how these are summarised with characteristics (Figure 1), which then flows into the differentiation of the four domains (Line 273-283).

Line 282-288: Providing examples of actual typology combinations would help the readers’ interpretability.

Line 308-312: As mentioned previously, the formatting and grammar from this point would be worth reviewing.

For example, multiple unnecessary clauses: “The Checklist, which can be completed in 5 to 10 minutes by an individual very familiar with the RPM program in question was to be used to collect sufficiently detailed semi-structured data about any given RPM program’s features and characteristics such that the information could then be used to establish the categorization of the RPM program.”

This could be: “The Checklist can be completed within 10 minutes by those familiar with the RPM program in question to collect detailed semi-structured data about the features and characteristics to establish the categorization of the RPM program”.

Line 316: Similar to the macros comment above, either expand why the developer features were used or provide a clear description in the appendices so the reader is aware of the process.

Discussion

Line 418-: The limitations are duly noted, however, detailing how community-based or LMIC programs may be underrepresented is key here. Tied to this, it may be fair to acknowledge that high-equity and high-integration models may not always be optimal depending on the context and environment.

Tying in a critique of the KTA model would also be appropriate in the limitations, for example, marginalising community expertise and resource and its usefulness in addressing structural inequities, which provides an opportunity to reflect on your approach using this in the present study.

General

Formatting needs some attention, for example, page numbers start on pg10, and Figure 3 is the first to appear on pg9.

**********

6. PLOS authors have the option to publish the peer review history of their article (what does this mean?). If published, this will include your full peer review and any attached files.

**Do you want your identity to be public for this peer review?**  If you choose “no”, your identity will remain anonymous but your review may still be made public.

For information about this choice, including consent withdrawal, please see our Privacy Policy.

Reviewer #1: No

**********

[NOTE: If reviewer comments were submitted as an attachment file, they will be attached to this email and accessible via the submission site. Please log into your account, locate the manuscript record, and check for the action link "View Attachments". If this link does not appear, there are no attachment files.]

**Figure resubmission:**

While revising your submission, we strongly recommend that you use PLOS’s NAAS tool (https://ngplosjournals.pagemajik.ai/artanalysis) to test your figure files. NAAS can convert your figure files to the TIFF file type and meet basic requirements (such as print size, resolution), or provide you with a report on issues that do not meet our requirements and that NAAS cannot fix.

After uploading your figures to PLOS’s NAAS tool - https://ngplosjournals.pagemajik.ai/artanalysis, NAAS will process the files provided and display the results in the "Uploaded Files" section of the page as the processing is complete. If the uploaded figures meet our requirements (or NAAS is able to fix the files to meet our requirements), the figure will be marked as "fixed" above. If NAAS is unable to fix the files, a red "failed" label will appear above. When NAAS has confirmed that the figure files meet our requirements, please download the file via the download option, and include these NAAS processed figure files when submitting your revised manuscript.

**Reproducibility:**

To enhance the reproducibility of your results, we recommend that authors of applicable studies deposit laboratory protocols in protocols.io, where a protocol can be assigned its own identifier (DOI) such that it can be cited independently in the future. Additionally, PLOS ONE offers an option to publish peer-reviewed clinical study protocols. Read more information on sharing protocols at https://plos.org/protocols?utm_medium=editorial-email&utm_source=authorletters&utm_campaign=protocols

# Author comment: Author response to Decision Letter 1

**DOI:** 10.1371/journal.pdig.0001595.r002

12 May 2026

Attachment Submitted filename: Response to Reviewers_08MAY2026.docx

# Peer review: Decision Letter 1

**DOI:** 10.1371/journal.pdig.0001595.r003

**Authors:** Hongxing Luo

6 Jul 2026

Design and development of a disease-agnostic remote patient monitoring typology and associated tools

PDIG-D-25-00742R1

Dear Dr. Abejirinde,

We are pleased to inform you that your manuscript 'Design and development of a disease-agnostic remote patient monitoring typology and associated tools' has been provisionally accepted for publication in PLOS Digital Health.

Before your manuscript can be formally accepted you will need to complete some formatting changes, which you will receive in a follow-up email from a member of our team.

Please note that your manuscript will not be scheduled for publication until you have made the required changes, so a swift response is appreciated.

IMPORTANT: The editorial review process is now complete. PLOS will only permit corrections to spelling, formatting or significant scientific errors from this point onwards. Requests for major changes, or any which affect the scientific understanding of your work, will cause delays to the publication date of your manuscript.

If your institution or institutions have a press office, please notify them about your upcoming paper to help maximize its impact. If they'll be preparing press materials, please inform our press team as soon as possible -- no later than 48 hours after receiving the formal acceptance. Your manuscript will remain under strict press embargo until 2 pm Eastern Time on the date of publication. For more information, please contact digitalhealth@plos.org.

Thank you again for supporting Open Access publishing; we are looking forward to publishing your work in PLOS Digital Health.

Best regards,

Hongxing Luo, M.D., Ph.D.

Section Editor

PLOS Digital Health

***********************************************************

**Additional Editor Comments (if provided):**

The Fig. 3 seems to appear before the Fig 1.

Please carefully check the numbering of all Figures, Tables and Supplements during the proofreading process.

**Reviewer Comments (if any, and for reference):**

Reviewer's Responses to Questions

**Comments to the Author**

1. If the authors have adequately addressed your comments raised in a previous round of review and you feel that this manuscript is now acceptable for publication, you may indicate that here to bypass the “Comments to the Author” section, enter your conflict of interest statement in the “Confidential to Editor” section, and submit your "Accept" recommendation.

Reviewer #1: All comments have been addressed

**********

2. Does this manuscript meet PLOS Digital Health’s publication criteria? Is the manuscript technically sound, and do the data support the conclusions? The manuscript must describe methodologically and ethically rigorous research with conclusions that are appropriately drawn based on the data presented.

Reviewer #1: Yes

**********

3. Has the statistical analysis been performed appropriately and rigorously?

Reviewer #1: Yes

**********

4. Have the authors made all data underlying the findings in their manuscript fully available (please refer to the Data Availability Statement at the start of the manuscript PDF file)?

The PLOS Data policy requires authors to make all data underlying the findings described in their manuscript fully available without restriction, with rare exception. The data should be provided as part of the manuscript or its supporting information, or deposited to a public repository. For example, in addition to summary statistics, the data points behind means, medians and variance measures should be available. If there are restrictions on publicly sharing data—e.g. participant privacy or use of data from a third party—those must be specified.

Reviewer #1: Yes

**********

5. Is the manuscript presented in an intelligible fashion and written in standard English?

PLOS Digital Health does not copyedit accepted manuscripts, so the language in submitted articles must be clear, correct, and unambiguous. Any typographical or grammatical errors should be corrected at revision, so please note any specific errors here.

Reviewer #1: Yes

**********

6. Review Comments to the Author

Please use the space provided to explain your answers to the questions above. You may also include additional comments for the author, including concerns about dual publication, research ethics, or publication ethics. (Please upload your review as an attachment if it exceeds 20,000 characters)

Reviewer #1: The authors have addressed or justified my review comments, with clear and substantive revisions to the methods, results, and discussion sections that now improve the clarity, methodological transparency, and interpretability of the manuscript. The final version is coherent, well-justified, and meets the expected standards for publication.

**********

7. PLOS authors have the option to publish the peer review history of their article (what does this mean?). If published, this will include your full peer review and any attached files.

**Do you want your identity to be public for this peer review?**  If you choose “no”, your identity will remain anonymous but your review may still be made public.

For information about this choice, including consent withdrawal, please see our Privacy Policy.

Reviewer #1: No

**********
